# The effect of mind-body exercise on the cervical spine mobility of people with neck discomfort: A protocol for systematic review and meta-analysis

**DOI:** 10.1097/MD.0000000000026112

**Published:** 2021-06-04

**Authors:** Xianhui Liao, Beihai Ge, Qiang Chen

**Affiliations:** a Department of Sports, Wuhan EQ & IQ School, Wuhan, Hubei; b Department of Neurology; c Department of Psychiatry, Guangxi Zhuang Autonomous Region Brain Hospital, Liuzhou, Guangxi, China.

**Keywords:** cervical spine mobility, meta-analysis, mind-body exercise, neck discomfort, protocol

## Abstract

**Background:**

With the development of the economy and society, the pace of in-person work has gradually accelerated, resulting in longer and more intense work hours. Long-term and high-intensity work can lead to considerable discomfort in people's cervical spines.

**Objectives:**

This study aims to explore the effect of mind-body exercise intervention on the cervical spine mobility of people with neck discomfort through meta-analysis.

**Methods:**

This study's researchers will search a total of 5 research databases for data retrieval: China National Knowledge Infrastructure (from 1979), Web of Science (from 1950), PubMed (from 1965), Cochrane (from 1991), and EBSCO (from 1949) (Date of retrieval: March 10, 2021). Two authors will independently search literature records, scan titles, abstracts, and full texts, collect data, and assess materials for risk of bias. Stata14.0 software will be used for the data analysis.

**Results:**

The current study is a systematic review and meta-analysis program with no results. Data analysis will be completed after the program has been completed.

**Discussion:**

There is potential evidence that exercise can have a positive effect on the cervical spine mobility of people with cervical spine discomfort. In addition, direct evidence of the benefits of mind-body exercise intervention may be more important.

**INPLASY Registration Number:**

INPLASY202140126.

## 1 Introduction

With the development of the economy and society, the pace of in-person work has gradually accelerated, resulting in longer and more intense work hours. The working methods of modern society are primarily different from the past due to electronic products. Long-term and high-intensity work can lead to considerable discomfort in people's cervical spines.

Neck discomfort is a common symptom of students and office workers and other people that can lead to clear dysfunction in neck movement.^[1]^ There can be many reasons for neck discomfort, such as maintaining the same posture for long periods of time,^[2]^ lack of sleep,^[3]^ and structural cervical lesions,^[4]^ etc. The harm to the body that neck discomfort causes does not appear within a short period.^[5]^ If neck discomfort is not resolved, it can cause dizziness,^[6]^ nausea,^[7]^ irritability,^[8]^ shoulder and neck pain,^[9]^ and other symptoms. Long-term maintenance of this state is detrimental to physical health and affects the mobility of the entire cervical spine.^[10]^ Cervical range of motion (CROM)^[11]^ refers to the movement of the cervical spine in the sagittal, coronal, and horizontal planes, and it includes forward flexion, extension, left and right lateral flexion, and left and right rotation. Each degree of freedom in the CROM is a good index for evaluating neck movement function because most neck diseases^[12,13]^ cause changes in cervical spine mobility. Clinically, CROM refers to the range of motion of the cervical spine, the cervical spine's left and right bends are 45 degrees each, and its left and right rotations are 45 degrees each. A normal cervical spine's range of motion includes 35 to 45 degrees of forward flexion and 35 to 45 degrees of backward extension.^[14]^ A limited CROM is detrimental to health.^[15]^

Mind-body exercise is a multi-modal exercise.^[16,17]^ Its typical characteristics are slow body movement, whole body stretching and relaxation, breathing control and mental concentration, and other structured forms of movement. Of course, exercises include many types, mind-body exercise is just one form of it. There are also many cases of intervention that use other exercise methods.^[18,19]^ When compared to other aerobic or resistance exercises,^[20,21]^ the advantages of mind-body exercise include its slow rhythm and stable intensity,^[16]^ which are conducive to long-term health development. Moreover, many research results have proven that mind-body exercise is beneficial to the treatment of chronic diseases.^[22]^ The primary forms of mind-body exercise include Tai Chi, Qigong, Baduanjin, Wuqinxi, and Yijinjing.^[23]^ It is also significant that mind-body exercise does not require the assistance of sports equipment,^[24]^ that its learning cost is low, and that its safety intensity is high, enabling it to be promoted on a large scale.^[25]^ This article aims to expand the sample size of randomized controlled experiments through meta-analysis,^[26]^ in order to explore the effect of mind-body exercise interventions on the cervical spine mobility of people with neck discomfort by providing feasibility opinions for treatment guidelines, clinicians, and related groups.

## 2 Methods

### 2.1 Registration

We conducted this systematic review in accordance with the guidelines for the Preferred Reporting Items for Systematic Reviews and Meta-analyses,^[27]^ and we completed the research registration on the INPLASY platform (Registration Number: INPLASY202140126).

### 2.2 Eligibility criteria

#### 2.2.1 Inclusion criteria

##### 2.2.1.1 Types of study

This study included select randomized controlled trials^[28]^ in peer-reviewed journals.

##### 2.2.1.2 Types of participants

This study included patients with cervical spine discomfort. The age of the population was ≥18 years. In addition, the population showed neck pain, soreness, stiffness, discomfort, restricted mobility, fatigue, shoulder, and back pain, dizziness, and other symptoms that did not involve the neck. Other than head discomfort, there were no neck symptoms caused by other diseases.

##### 2.2.1.3 Types of intervention

The intervention period needed to be greater than or equal to 4 weeks. Subjects were generally divided according to 2 types of intervention methods:

1. the intervention group used a single method of mind-body exercise, or Taijiquan, Baduanjin, Qigong, Wuqinxi, or Yijinjing, and the control group used no other measures or acupuncture, traction, or massage; or
2. the intervention group used mind-body exercise, or Taijiquan, Baduanjin, Qigong, Wuqinxi, or Yijinjing, with no other measures or acupuncture, traction, or massage, and the control group used no other measures or acupuncture, traction, or massage.

##### 2.2.1.4 Types of outcome measures

This study aimed to evaluate the effect of mind-body exercise on the cervical spine mobility of patients with cervical spine discomfort. After a preliminary search of data, we determined that cervical spine mobility is the most effective and direct indicator of cervical spine motor function. It has gradually become the primary measure for evaluating cervical spine function and cervical spine injury. It is also an important reference index for assessing degrees of damage, diagnosing and identifying neck diseases, curative effect evaluations, and prognostic analyses.^[13]^ There are many kinds of measurements of cervical spine mobility, including visual inspection,^[29]^ tape measures,^[30]^ inclinometer measurement,^[31]^ electromagnetic motion analysis,^[32]^ and other techniques using electronic measuring instruments.^[33]^ In order to consider the convenience and accuracy of actual measurement tools, this study used the following tools: Multi-Cervical Unit^[34]^ and CROM for a measure of cervical spine mobility.^[35]^

### 2.3 Exclusion criteria

This study's exclusion criteria included

1. research with incomplete data;
2. the use of cervical spine mobility measurement tools that were not Multi-Cervical Unit or CROM;
3. the presence of medical contraindications, such as fractures and local tumors; and
4. patients who were participating in other clinical trials.

### 2.4 Information sources and searches

Five research databases were used for retrieval in this study: China National Knowledge Infrastructure (from 1979), Web of Science (from 1950), PubMed (from 1965), Cochrane (from 1991), and EBSCO (from 1949) (Date of retrieval: March 10, 2021). In addition, the references for the included literature were searched manually. This study used 2 sets of keywords:

1. mind-body exercise, Taiji, Taijiquan, Baduanjin, Qigong, Wuqinxi, and Yijinjing; and
2. cervical spondylosis, neck pain, cervical pain, and neck discomfort.

The search formula was based on the EBSCO database search formula (Example: SU = neck pain or cervical spondylosis or cervical pain or neck discomfort and SU = mind-body exercise or Taiji or Taijiquan or Baduanjin or Qigong or Wuqinxi or Yijinjing). The literature search was carried out by the author (LXH) and by another collaborator (GBH) to ensure accuracy during data retrieval.

### 2.5 Data collection process

Each article was extracted by two independent researchers (LXH, GBH) and converted into the following standard forms:

1. descriptive statistics that included the first author of the article, the year of publication, and the health of the subjects; diagnostic criteria, sample size, and number of tested subjects; duration of disease, average age, and age range of the tested subjects; training time per session, number of training sessions per week, and total number of training weeks; forms of mind-body exercise and experimental group and control group interventions; and primary measurement results, adverse events, and follow-up; and
2. quantitative data that included random allocations of subjects; the average ± standard deviation of the baseline data of the experimental group and the control group; and the average ± standard deviation of the experimental group and the control group after intervention. Finally, a third researcher (CQ) checked and evaluated the effects of interventions after verifying the information to avoid mistakes caused by human error.

### 2.6 Risk of bias across studies

In order to independently evaluate the methodology of the included studies, 2 authors (LXH, GBH) used a modified Physiotherapy Evidence Database (PEDro) Scale to evaluate the included literature. The 2 authors independently evaluated the literature. If they encountered differences, they discussed and resolved their analyses. If they could not reach an agreement on these differences, the third evaluator (CQ) was asked to evaluate the issue so they could finally reach an agreement.

### 2.7 Data synthesis and additional analyses

By using Stata 14.0 software, all the outcome indicators included in the literature were found to be continuous variables. The mean ± standard deviation was selected for statistics. Because the outcome indicators were measured by different tools, standard mean difference was selected for the combined effect size, and all the outcome indicators were tested for heterogeneity. Using *P* value and *I*^2^ for heterogeneity statistics, there was no heterogeneity between the studies if *P* > .10, and there was heterogeneity between the studies if *P* < .10. By comparison, there was a low and acceptable degree of heterogeneity between the studies if *I*^2^ < 25%; a low to medium degree of heterogeneity between the studies if 25% < *I*^2^ < 50%; a medium to high degree of heterogeneity between the studies if 50% < *I*^2^ < 75%; and a high degree of heterogeneity between the studies if *I*^2^ > 75%. Subgroup and sensitivity analyses were used to explore the sources of heterogeneity in outcome indicators, combined effect size and publication bias tests were used to calculate the effect size and risk of bias in the publications, and forest and funnel diagrams were drawn.

## 3 Discussion

### 3.1 Summary of evidence

The research included in this review solely involved randomized controlled trials studies that used combined effect size, subgroup analysis, sensitivity analysis, publication bias tests, and other methods to evaluate the effect of mind-body exercise on the cervical spine mobility of people with neck discomfort.

### 3.2 Comparisons with previous studies

This study's search of published data revealed no meta-analyses of studies on the influence of mind-body exercise on the cervical spine mobility of people with neck discomfort. Therefore, we conducted additional searches from the perspective of exercise and found that existing studies have reached a cautious conclusion: yoga can increase the cervical spine mobility of patients with chronic cervical spondylosis, but the specific strength of evidence requires subsequent research and exploration.^[19]^ There is also evidence that stretching exercises can directly affect the range of motion of the cervical spine and that passive hamstring stretching exercises can directly affect the range of motion and balance of the cervical spine.^[36]^ This evidence indicates that exercise may improve the cervical spine mobility of patients with cervical spondylosis.

Although there is potential evidence that exercise can have a positive effect on the cervical spine mobility of people with cervical spine discomfort, direct evidence of the benefits of exercise intervention may be more important. Due to the limited number of relevant and existing studies, there is a need to explore related evidence at a later period.

## Acknowledgments

The authors would like to acknowledge the participants for taking part in the study.

Supporting information: Seeing Search strategy, Supplemental Content, which shows the search strategy of this study.

## Author contributions

**Conceptualization:** Xianhui Liao, Beihai Ge, Qiang Chen.

**Data curation:** Xianhui Liao, Beihai Ge.

**Funding acquisition:** Beihai Ge.

**Methodology:** Xianhui Liao, Beihai Ge, Qiang Chen.

**Project administration:** Xianhui Liao, Qiang Chen.

**Resources:** Xianhui Liao, Beihai Ge, Qiang Chen.

**Supervision:** Qiang Chen.

**Writing – original draft:** Xianhui Liao, Beihai Ge, Qiang Chen.

**Writing – review & editing:** Xianhui Liao, Beihai Ge, Qiang Chen.

## Supplementary Material

Supplemental Digital Content
