# Supplementary material for: The effect of mind-body exercise on the cervical spine mobility of people with neck discomfort: A protocol for systematic review and meta-analysis
Source: Medicine (Baltimore). 2021 Jun 4;100(22):e26112. doi: 10.1097/MD.0000000000026112 (PMC8183790; doi:10.1097/MD.0000000000026112)
Supplement: Supplemental Digital Content [file medi-100-e26112-s001.docx]

**Search Strategy**

**Pubmed**

(neck pain[Mesh Terms] OR cervical spondylosis[Mesh Terms] OR cervical pain[Mesh Terms] OR neck discomfort[Mesh Terms]) AND (mind-body exercise[Mesh Terms] OR taichi[Mesh Terms] OR taijiquan[Mesh Terms] OR baduanjin[Mesh Terms] OR qigong[Mesh Terms] OR wuqinxi[Mesh Terms] OR yijinjing[Mesh Terms]）

Form 1965

**Cochrane library**

#1: neck pain

#2: cervical spondylosis

#3: cervical pain

#4: neck discomfort

#5: mind-body exercise

#6: taichi

#7: taijiquan

#8: baduanjin

#9: wuqinxi

#10: yijinjing

#11: qigong

(#1 OR #2 OR #3 OR #4) AND (#5 OR #6 OR #7 OR #8 OR #9 OR #10 OR #11)

From 1991

**WOS (Web of science)**

TS = (neck pain OR cervical spondylosis OR cervical pain OR neck discomfort) AND TS = (mind-body exercise OR taichi OR taijiquan OR baduanjin OR qigong OR wuqinxi OR yijinjing)

From 1950

**EBSCO**

SU = (neck pain OR cervical spondylosis OR cervical pain OR neck discomfort) AND SU = (mind-body exercise OR taichi OR taijiquan OR baduanjin OR qigong OR wuqinxi OR yijinjing)

From 1949

**CNKI（China National Knowledge Infrastructure）**

SU = neck pain + cervical spondylosis + cervical pain + neck discomfort AND SU = mind-body exercise + taichi + taijiquan + baduanjin + qigong + wuqinxi + yijinjing

Form 1979
